# Tropism and Induction of Cytokines in Human Embryonic-Stem Cells-Derived Neural Progenitors upon Inoculation with Highly- Pathogenic Avian H5N1 Influenza Virus

**DOI:** 10.1371/journal.pone.0135850

**Published:** 2015-08-14

**Authors:** Kidsadagon Pringproa, Ruttachuk Rungsiwiwut, Rachod Tantilertcharoen, Reunkeaw Praphet, Kamthorn Pruksananonda, Wolfgang Baumgärtner, Roongroje Thanawongnuwech

**Affiliations:** 1 Department of Veterinary Biosciences and Veterinary Public Health, Faculty of Veterinary Medicine, Chiang Mai University, Chiang Mai, Thailand; 2 Human Embryonic Stem Cell Research Center, Reproductive Medicine Unit, Department of Obstetrics and Gynecology, Faculty of Medicine, Chulalongkorn University and King Chulalongkorn Memorial Hospital, Bangkok, Thailand; 3 Veterinary Diagnostic Laboratory, Faculty of Veterinary Science, Chulalongkorn University, Bangkok, Thailand; 4 Institute Product Quality and Standardization, Maejo University, Chiang Mai, Thailand; 5 Department of Pathology, University of Veterinary Medicine Hannover, Hannover, Germany; 6 Department of Pathology, Faculty of Veterinary Sciences, Chulalongkorn University, Bangkok, Thailand; The Chinese University of Hong Kong, HONG KONG

## Abstract

Central nervous system (CNS) dysfunction caused by neurovirulent influenza viruses is a dreaded complication of infection, and may play a role in some neurodegenerative conditions, such as Parkinson-like diseases and encephalitis lethargica. Although CNS infection by highly pathogenic H5N1 virus has been demonstrated, it is unknown whether H5N1 infects neural progenitor cells, nor whether such infection plays a role in the neuroinflammation and neurodegeneration. To pursue this question, we infected human neural progenitor cells (hNPCs) differentiated from human embryonic stem cells *in vitro* with H5N1 virus, and studied the resulting cytopathology, cytokine expression, and genes involved in the differentiation. Human embryonic stem cells (BG01) were maintained and differentiated into the neural progenitors, and then infected by H5N1 virus (A/Chicken/Thailand/CUK2/04) at a multiplicity of infection of 1. At 6, 24, 48, and 72 hours post-infection (hpi), cytopathic effects were observed. Then cells were characterized by immunofluorescence and electron microscopy, supernatants quantified for virus titers, and sampled cells studied for candidate genes.The hNPCs were susceptible to H5N1 virus infection as determined by morphological observation and immunofluorescence. The infection was characterized by a significant up-regulation of TNF-α gene expression, while expressions of IFN-α2, IFN-β1, IFN-γ and IL-6 remained unchanged compared to mock-infected controls. Moreover, H5N1 infection did not appear to alter expression of neuronal and astrocytic markers of hNPCs, such as β-III tubulin and GFAP, respectively. The results indicate that hNPCs support H5N1 virus infection and may play a role in the neuroinflammation during acute viral encephalitis.

## Introduction

Influenza caused by highly-pathogenic avian H5N1 virus has been one of the most important zoonotic viral infections of humans during the last decade [1–3], with human fatality rates more than 50 percent in some areas of 15 affected countries [4], where outbreaks continue. Influenza viruses belong to the family *Orthomyxoviridae*, of which the H5N1 type has a broad range of hosts [1,5]. Although H5N1 naturally infects poultry and wild birds, transmission occurs to mammalian species, including humans [6–9]. H5N1-infected humans often develop severe clinical respiratory and gastrointestinal symptoms and signs; in some cases, symptoms of the central nervous system (CNS) ensue [10–13]. Infection of the CNS by H5N1 may be severe, causing encephalopathy and other serious neurological complications and sequelae [14–16]. Recent reports found that mice experimentally infected with H5N1 virus developed encephalitic lesions during the acute phase, which was associated with a degree of neuronal degeneration and necrosis. Interestingly, CNS inflammation has not generally been observed in the chronic phase of infection, despite the detection of virus in such lesions [17]. Furthermore, Jang *et al*. (2009) reported protein aggregation mediated by the influenza virus within degenerated and necrotic neurons. These findings suggest the hypothesis that pathogenic influenza virus might induce a “hit and run mechanism”, in which virus infection triggers a cytokine storm resulting in acute CNS inflammation, with consequent chronic Parkinson-like disease, encephalitis lethargica, or other neurodegenerative diseases. Nevertheless, the exact pathologic mechanism(s) of H5N1-induced encephalopathy remains unclear.

Neurogenesis in the adult CNS is regulated by the neural stem/progenitor cells residing in the subventricular zone (SVZ) of the lateral ventricles, and subgranular zone (SGZ) of the hippocampus [18,19]. This neurogenesis was shown to be de-regulated by infection with neurovirulent viruses causing Borna disease and Varicella-zoster [20,21]. Thus, it would be essential to determine whether infection by H5N1 virus occurs in neural progenitor cells, and if so, whether such infection plays a role in the pathogenesis of H5N1-induced encephalopathy. In order to shed light on these questions relevant to the pathogeneses of influenza virus-induced neuropathology, we infected human neural progenitor cells (hNPCs) with H5N1 avian influenza virus and examined the resulting virus-cell interactions, the induction of cellular mediators, and the phenotypic characterizations of hNPCs following H5N1 virus infection *in vitro*.

## Materials and Methods

### Differentiation of human embryonic stem cells

The human embryonic stem cell (hESC) line BG01 (WiCell Research Institute, Madison, WI, USA) was cultured and maintained as previously described [22]. Briefly, BG01 cells were plated on Mitomycin C (Sigma Aldrich, St. Louis, MO, USA)-inactivated human foreskin fibroblasts (HFFs; CRL 2429; American Type Culture Collection, Manassas, VA, USA) in a culture medium consisting of knockout Dulbecco’s modified Eagle’s medium (KO-DMEM) supplemented with 20% knockout serum replacement, 1% Glutamax, 1% non-essential amino acids, 0.1 mM 2-mercaptoethanol, 1% penicillin-streptomycin, and 8 ng/mL basic fibroblast growth factor (bFGF; all purchased from Invitrogen, Carlsbad, CA, USA). The hESCs were differentiated into hNPCs via embryoid body (EB) formation as described previously [22]. The hNPCs were cultured on Matrigel-coated dishes in a culture medium consisting of neurobasal medium, 1% Glutamax, 1% penicillin-streptomycin, 1% N2, 2% B-27, 20 ng/mL bFGF, and 20 ng/mL epidermal growth factor (EGF; all from Invitrogen, Carlsbad, CA, USA), and used for further studies. Characterization of the hNPCs was done by immunofluorescence and reverse transcriptase polymerase chain reaction (RT-PCR), as described below.

### Virus propagation and infection

H5N1 avian influenza virus (A/Chicken/Thailand/CUK2/04), isolated from chickens in Thailand during the early 2004 outbreak, was propagated in Madin-Darby Canine Kidney (MDCK) cells, as previously described [23,24]. The hNPCs were infected with either H5N1, or ultraviolet (UV)-inactivated H5N1 virus at a multiplicity of infection (MOI) of 1. Inactivation of H5N1 virus by UV light was done at a distance of 25 cm for 1 hour as previously described [25], and its infectivity was verified by the presence of cytopathic effects (CPEs), and immunofluorescene for influenza A antigen, as described below. Virus propagation and infection were conducted in Biosafety Laboratory Level 3, at the Center for Emerging and Re-emerging Infectious Diseases of Chulalongkorn University, Bangkok, Thailand. Briefly, after 1 hour of virus absorption at room temperature (RT), the inoculum was removed, the cultures were washed once with medium without fetal bovine serum (FBS), and replaced with the complete medium, as mentioned above. At 6, 24, 48 and 72 hpi, the cells and supernatants in 24 wells-microtiter plates were collected, respectively, for RNA isolation and viral growth kinetic study. The virus-infected cells in 96 wells-microtiter plates were observed and graded for the CPEs under a light microscope compared to the medium alone infected groups, which served as mock-infected control. Thereafter, they were fixed and immunostained with specific markers, as described below. The data were obtained from the experiments performed in triplicate.

### Viral growth kinetic study

Quantization of viral growth kinetics was done in MDCK cells, as previously described [26]. In brief, supernatants from the H5N1-infected, or UV-inactivated H5N1- infected hNPCs at different time points (6, 24, 48 and 72 hours-post infection; hpi) were titrated, and inoculated onto a monolayer of MDCK cells. After 1 hour of virus infection, unbound viruses were discarded and the cells were maintained with a defined medium under standard culture conditions (5% CO_2_, 37°C). The CPEs were observed at day 3-post infection under an inverted microscope (Olympus IX-70, Olympus Corporation, Tokyo, Japan). The viral titers were reported as 50% tissue culture infectious dose (TCID_50_)/mL of culture supernatants.

### Transmission electron microscopy (TEM)

To study the cytopathology of H5N1-infected hNPCs, cells in 6-well microtiter plate were infected by a 1 MOI of H5N1 virus. At 24 hpi, cells were fixed in 2.5% glutaraldehyde for 48 hours, washed three times with phosphate buffer saline (PBS), and post fixed in 2% osmium tetroxide for 1 hour at RT. After being serially dehydrated in ethanol, the cells were incubated in propylene oxide and embedded in resin (EM-bed 812, Electron Microscope, Washington, USA). Semi-thin sections were cut and stained with toluidine blue for light microscope examination. Thereafter, ultra-thin sections were cut, mounted on copper grid, and electron contrasted with uranil acetate and lead citrate. The grids were examined and photos were taken under a JEM-2200FS transmission electron microscope (JEOL ltd., Tokyo, Japan).

### Immunofluorescence

Immunostaining of hNPCs prior to and following H5N1 virus infection were done in 96 wells-microtiter plates, as previously described [25]. Briefly, the cultures were fixed with 4% paraformaldehyde for 15 min at RT, and treated with 0.25% Triton X-100 in PBS (0.25% PBST) for 15 min. Then, the cells were incubated with 1% bovine serum albumin (BSA) in PBST for 30 min at RT, followed by incubation with specific primary antibodies diluted with 1% BSA in 0.25% PBST at 4°C, overnight. The primary antibodies used to characterize the hNPCs with or without virus infections were mouse monoclonal anti-Nestin (1:200; GeneTex, Irvine, CA, USA), mouse monoclonal anti-Sox1 (1:200; Millipore Corporation, Billerica, MA, USA), mouse monoclonal anti-glial fibrillary acidic protein (GFAP; 1:100; Millipore Corporation, Billerica, MA, USA), mouse monoclonal anti- III tubulin (1:100; Millipore Corporation, Billerica, MA, USA), mouse monoclonal anti-nucleoprotein of influenza A virus (1:400; clone HB-65, American Type Culture Collection, VA, USA), and rabbit polyclonal anti-Pax6 (1:200; R&D Systems, Minneapolis, MN, USA). The secondary antibodies were incubated for 45 min at RT with one of the following antibodies: Cy3–conjugated goat anti-mouse, FITC–conjugated goat anti-mouse and FITC-conjugated goat anti-rabbit antibodies (all from Jackson ImmunoResearch, Suffolk, UK), at a dilution of 1:200. The nuclei were counterstained using bisbenzimide (0.01% in ethanol, Sigma Aldrich, St. Louis, MO, USA) for 10 min at RT. The cultures were analyzed under an inverted fluorescent microscope. Average cell number per high power field was analysed, and the percentage of cells positive for specific markers was determined, as previously described [25].

### RT-PCR and quantitative RT-PCR

The total RNA of H5N1-infected and mock-infected hNPCs was extracted and determined using Nucleospin RNA II (Machery-Nagel GmbH, Dauren, Germany), as suggested by the manufacturer. The cDNA was synthesized from 500 ng total RNA with poly (dT) primers and high capacity cDNA reverse transcriptase kits (Applied Biosystems, CA, USA). The oligonucleotide primers genes used in this study were listed in Table 1. For Pax6 and Sox1 genes, conventional PCR was performed at the conditions as follows: 95°C for 2 min, 35 cycles of 95°C for 30 sec, 55°C for 30 sec, 72°C for 1 min, and final extension at 72°C for 2 min. The PCR products were analyzed by the electrophoresis, and specific bands were observed under the UV illuminator.

**Table 1:** Summary of primers used for RT-PCR and quantitative RT-PCR.

Genes	Sequence (5’-3’)		Position	Acc. no.
Sox1	Forward	CAATGCGGGGAGGAGAAGTC	1914–2361	NM_005986.2
	Reverse	CTCCTCTGGACCAAACTGTG		
Pax6	Forward	CAGCTCGGTGGTGTCTTTG	18–254	XM_005252958.2
	Reverse	AGTCGCTACTCTCGGTTTA		
IFN-α2	Forward	ACCTTTGCTTTACTGGTGGCC	78–179	NM_000605.3
	Reverse	ATCTGTGCCAGGAGCATCAAG		
IFN-β1	Forward	TCCTGTGGCAATTGAATGG	197–325	NM_002176.2
	Reverse	AATAGCGAAGATGTTCTGGAG		
IFN-γ	Forward	TCCCATGGGTTGTGTGTTTA	912–1109	NM_000619.2
	Reverse	AAGCACCAGGCATGAAATCT		
TNF-α	Forward	AGCCCATGTTGTAGCAAACC	442–572	NM_000594.3
	Reverse	TGAGGTACAGGCCCTCTGAT		
IL-6	Forward	GAACTCCTTCTCCACAAGCG	119–238	NM_000600.3
	Reverse	GCGGCTACATCTTTGGAATC		
GFAP	Forward	CCCACTCTGCTTTGACTGAGC	2828–2942	NM_002055.4
	Reverse	CCTTCTTCGGCCTTAGAGGG		
Nestin	Forward	AAGAGAACCTGGGAAAGGGAGAGT	2892–3025	NM_006617.1
	Reverse	TTCCTGAGCCAGTTCTTGGTCCTT		
β-III tubulin	Forward	CAACAGCACGGCCATCCAGG	1230–1413	NM_006086.3
	Reverse	CTTGGGGCCCTGGGCCTCCGA		
GAPDH	Forward	GTCAAGGCTGAGAACGGGAA	366–504	NM_002046.5
	Reverse	AAATGAGCCCCAGCCTTCTC		

Acc.no.: GeneBank accession number, Sox1: Sex determining region Y-box 1, Pax6: Paired box protein Pax-6, TNF: tumor necrosis factors, IFN: interferon, IL: interleukin, GFAP: glial fibrillary acidic protein, GAPDH: glyceraldehyde 3-phosphate dehydrogenase

To quantify the expression of cytokines, and specific genes for NPC differentiation, real time PCR (SYBR green selected master mix, Life Technologies, CA, USA) was performed on the ABI7300 thermo cycler (Applied Biosystems, CA, USA) in a total reaction volume of 20 μL. The PCR conditions were 95°C for 10 min, followed by 40 cycles of 95°C for 15 sec, 60°C for 30 sec, and 72°C for 30 sec. The threshold cycles (Ct) of all genes were used for the calculation of gene expression by the 2^-ΔΔCT^ method [27] normalized to that of GAPDH gene at the corresponding time points compared to mock-infected groups. The data were obtained from triplicate wells from at least two independent experiments, and shown as mean fold changes ± standard errors.

### Statistical analysis

The statistical analyses were accomplished using GraphPad Prism 5 (GraphPad Inc., La Jolla, CA, USA). Either t-test or one-way analysis of variance (ANOVA) of mean and median, followed by Tukey’s *post hoc* test, was performed according to the data types. Statistical significance was designated as *p* ≤ 0.05.

## Results

### Differentiation of human embryonic stem cells

**Fig 1:**
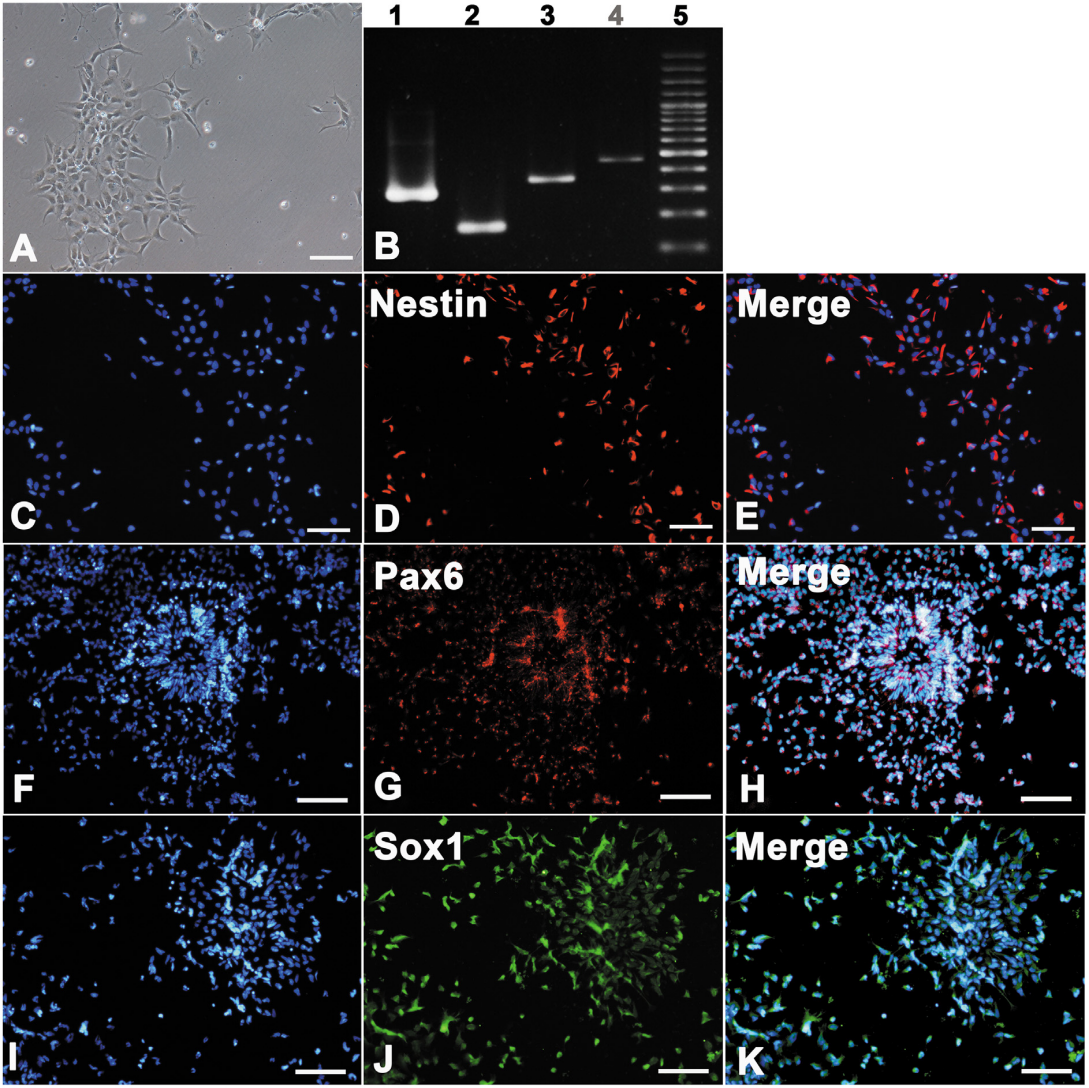
The immunostaining and mRNA expression of specific genes of hNPCs after 7 days *invitro*. Human NPCs displayed a bi- to multipolar morphology (**A**). The mRNA expression of hNPCs by RT-PCR (**B**) revealed strong positivity for Nestin (lane 2) and Pax6 (lane 3), slight positivity for Sox1 (lane 4) genes, compared to that of GAPDH gene (Lane 1). Characterization of the cells with antibodies against Nestin, Pax6, and Sox1 revealed more than 85% were immunostaining positive for the Nestin (**C-E**), Pax6 (**F-H**) and Sox1 (**I-K**) antibodies. Scale bar ≈ 100 μm.

Upon virus infection on day 7, the hNPCs (differentiated from hESCs) displayed morphology of spindle, bi- to multi-branch processes (Fig 1A). The mRNA expressions of Nestin, Pax6 and Sox1 genes indicated their neural progenitor phenotypes (Fig 1B). Immunophenotypic characterization of the hNPCs revealed that more than 85% of the cells expressed characteristic markers specific for neural progenitor phenotypes, including Nestin (Fig 1C–1E), Pax6 (Fig 1F–1H) and Sox1 (Fig 1I–1K).

### Cytopathology of hNPCs upon H5N1 infection

**Fig 2:**
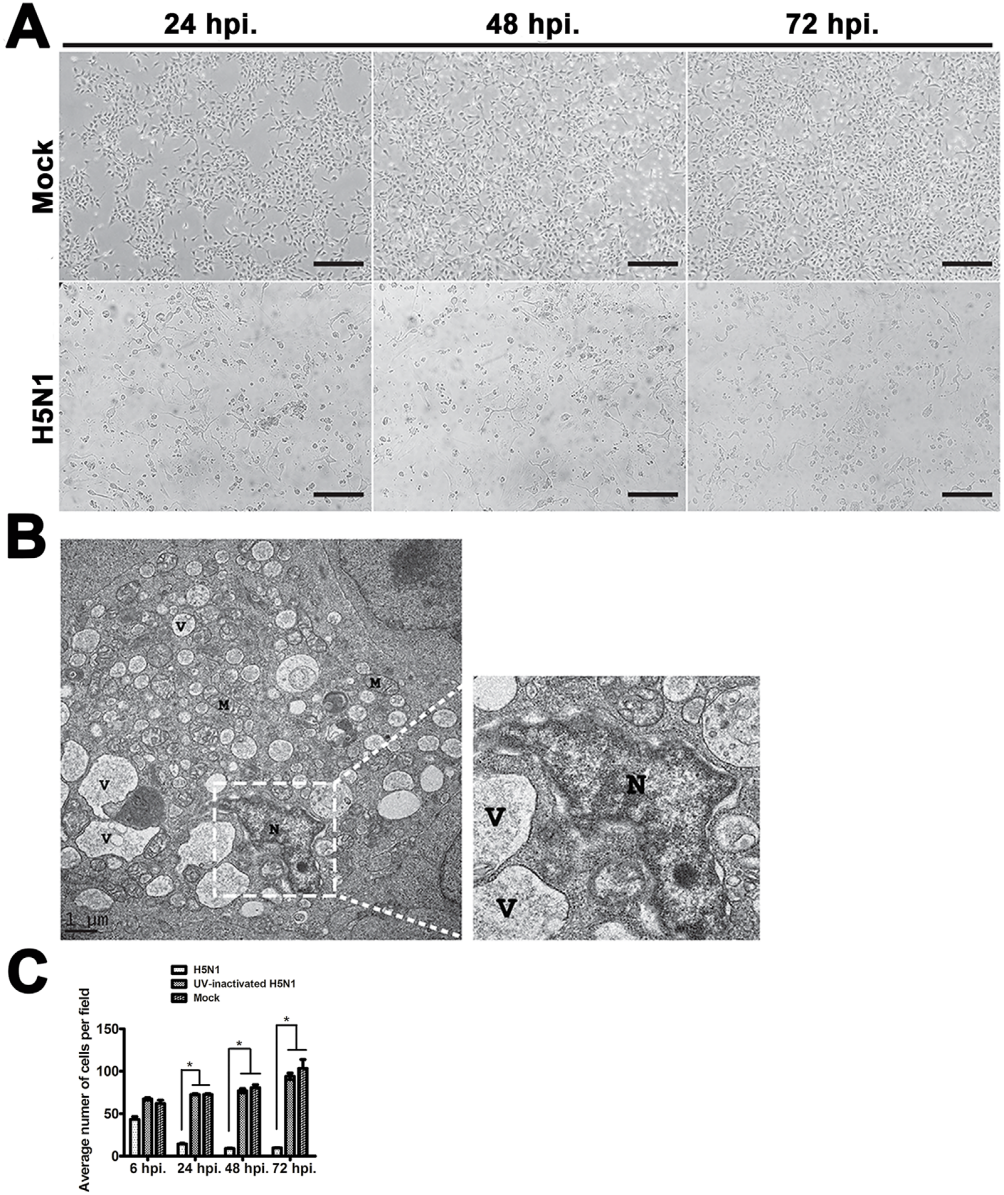
The cytopathic effects (CPEs), electron micrographs, and average cell number of hNPCs following H5N1 virus infection. At 24 hpi, 48 hpi, and 72 hpi, H5N1-infected hNPCs revealed the pronounced CPEs, such as cell rounding up, vacuolation, short processes of the cells, and cell detachment, while no CPEs were observed in the mock-infected control cells (**A**). Transmission electron micrographs (**B)** showed the changes in hNPC induced by H5N1 virus by shrinkage and condensation of the nucleus and chromatin, disruption of the cytoplasmic membrane and increased in cellular vacuolation (N = nucleus; V = vacuolation; M: mitochondria). The detachment of H5N1-infected hNPCs was observed by the significant decreased of average cell number per field at 24–72 hpi compared to the UV-inactivated H5N1- and mock-infected controls (**C**). Scale bar in A ≈100 μm, scale bar in B ≈ 1μm.

Infection of hNPCs with H5N1 virus displayed pronounced CPEs characterized by rounding up of cells, single cell necrosis, and detachment of the degenerated cells from the well surface (Fig 2A). The CPEs of infected cultures were up to 50% at 48 hpi, and 70% at 72 hpi (data not shown). Transmission-electron micrography of H5N1-infected hNPCs revealed shrinkage with condensation of the nuclear chromatin, and increased vacuolation in the cytoplasm (Fig 2B). Moreover, disruption of the cell and nuclear membranes was noticed. The detachment of H5N1-infected hNPCs was indicated by an observed significant decrease of average cell number per field at 24–72 hpi compared to the UV-inactivated H5N1-infected cells or mock-infected controls (Fig 2C).

### hNPCs support H5N1 virus infection

**Fig 3:**
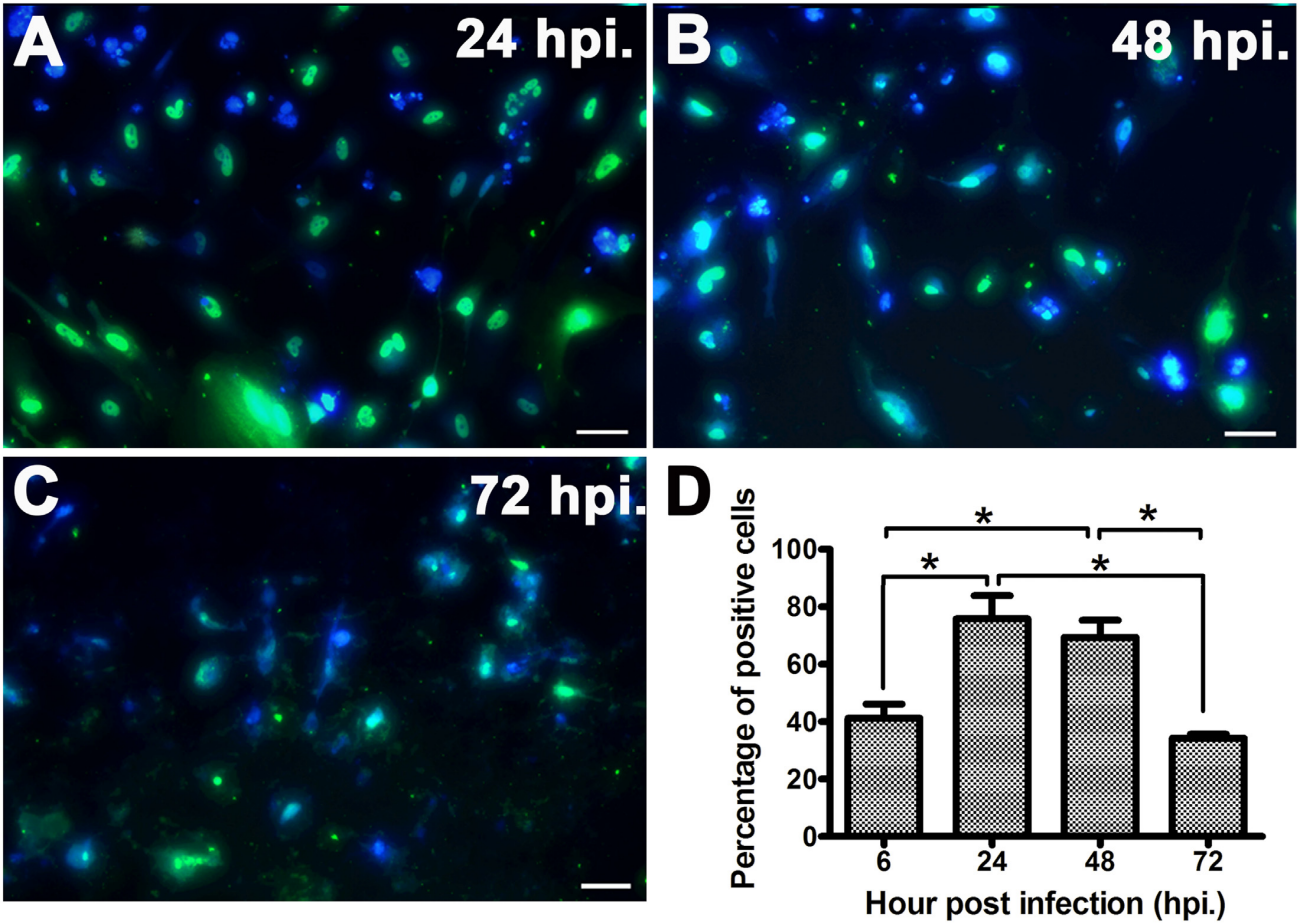
Immunostaining characterization and percentage of H5N1-positive cells of hNPCs following virus infection. Virus antigens were distributed within the cytoplasm and the nucleus of the infected cells (**A-C**), with peak percentage of virus-positive cells about 75% at 24 hpi, then declined to about 30% at 72 hpi (**D**). Scale bar in A-C ≈ 30 μm. Data represented are the mean ± standard error. Asterisks indicated statistically significant differences (*p*-value<0.05).

**Fig 4:**
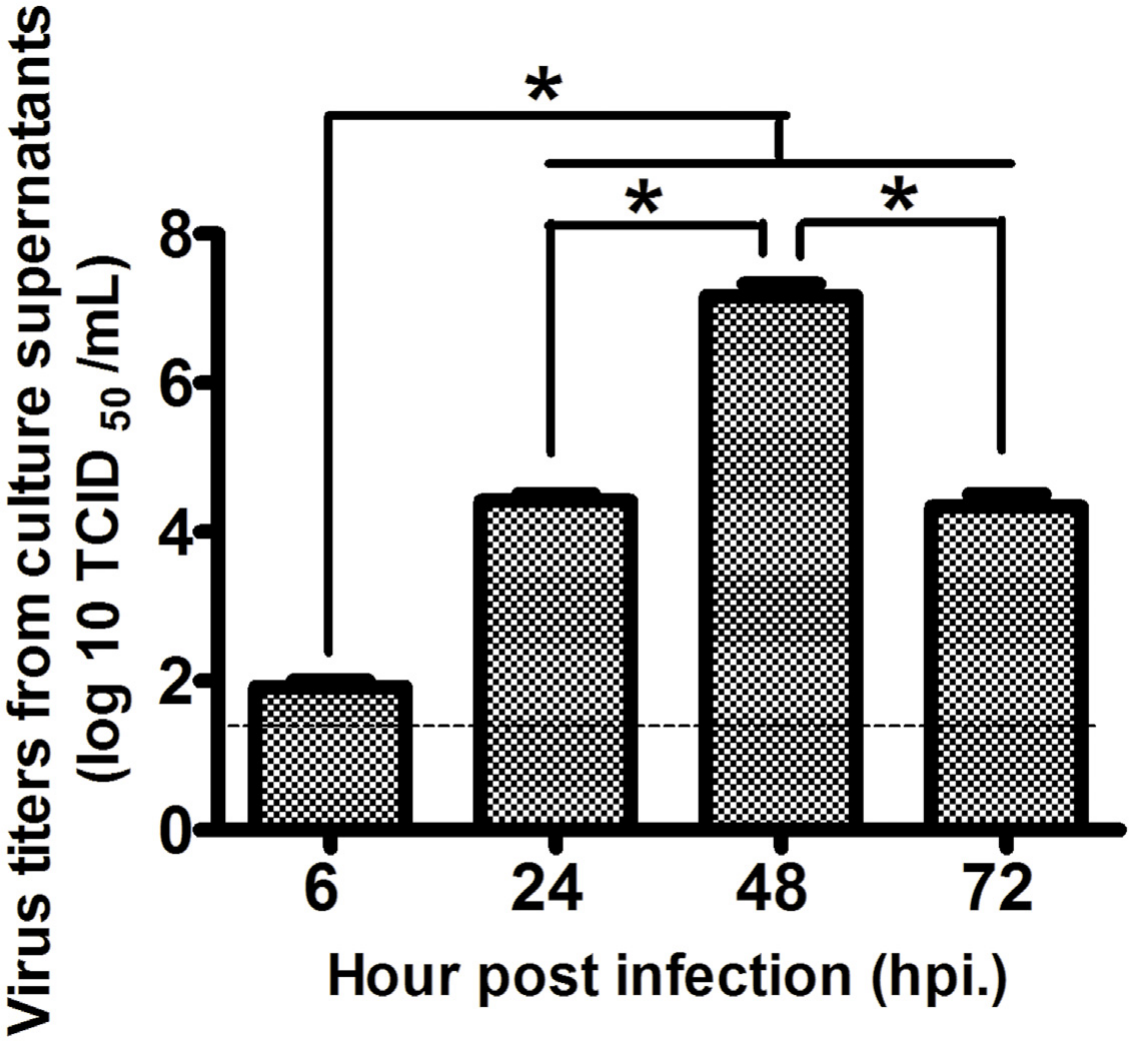
The virus kinetic study from the supernatants of H5N1-infected hNPCs at different time points post infection. The hNPCs supported H5N1 virus infection as determined by a statistical significant increase of virus titers from 10^2^ TCID_50_/mL at 6 hpi, which reached a maximum titer of 10^7^ TCID_50_/mL at 48 hpi. It subsequently declined to about 10^4^ TCID_50_/mL at 72 hpi. Results were obtained from three separate experiments. Data represented are the mean ± standard error. Asterisks indicated statistically significant differences (*p*-value<0.05). Dotted line represents detection limit of the assay.

Virus antigens were distributed within the cytoplasm and the nucleus of the infected hNPCs (Fig 3A–3C), with a peak percentage of virus-positive cells of about 75% at 24 hpi, then declining to about 30% at 72 hpi (Fig 3D). Quantification of progeny virus in the TCID_50_ assay of supernatants after viral growth revealed that hNPCs support H5N1 virus infection, as defined by an increase in viral titer of 10^2^ TCID_50_/mL at 6 hpi to 10^7^ TCID_50_/mL at 48 hpi, before declining to about 10^4^ TCID_50_/mL at 72 hpi (Fig 4).

### Cytokine gene expression of hNPCs following H5N1 infection

**Fig 5:**
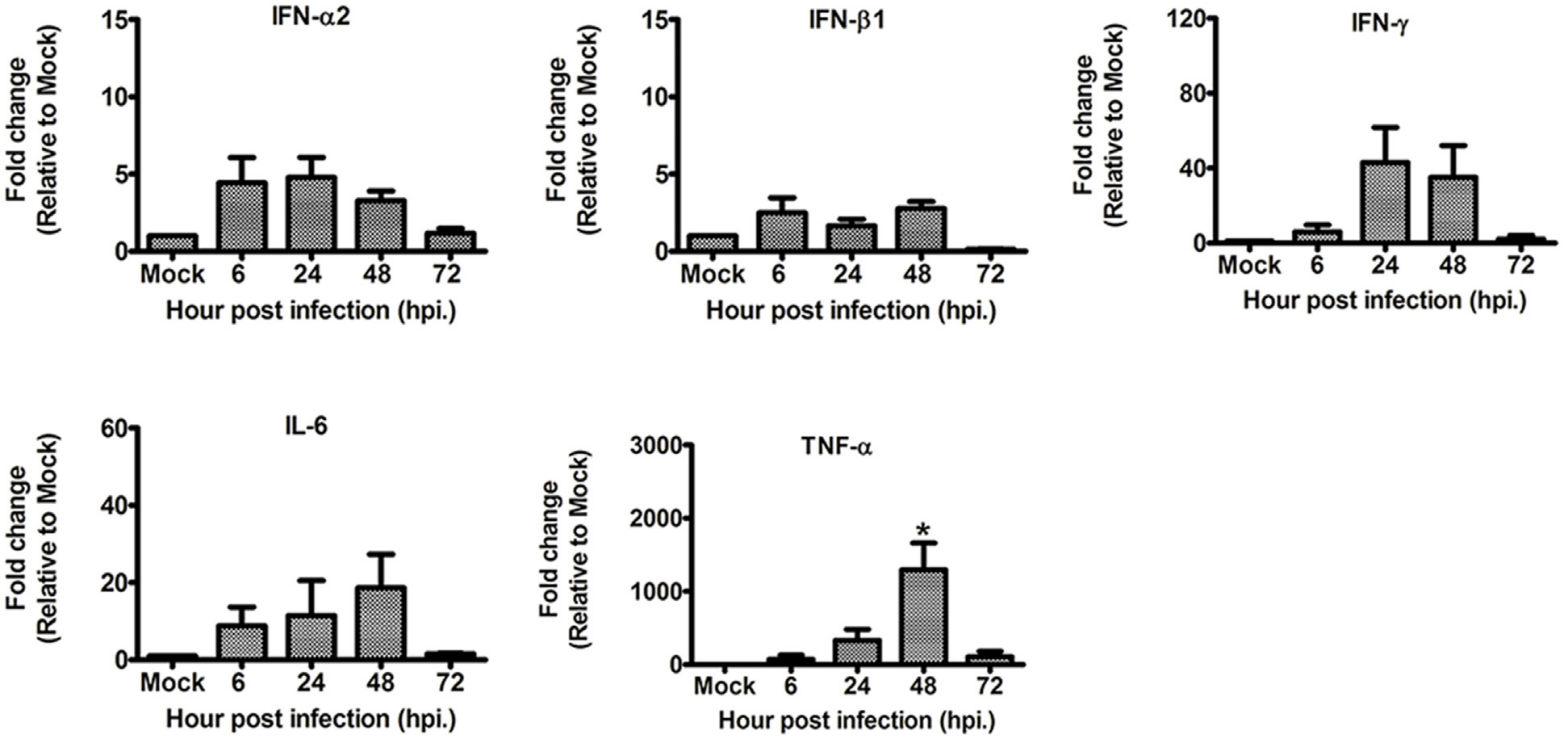
The expression of the mRNA levels of IFN-α2, IFN-β1, IFN-γ, TNF-α, and IL-6 in the hNPCs following infection with and without H5N1 virus by quantitative RT-PCR. The mRNA expressions of IFN-α2, IFN-β1, IFN-γ, and IL-6 were not significantly difference, while the mRNA level expression of TNF-α was observed to be significantly up-regulated at 48 hpi, compared to that of the control groups. Data are expressed as mean fold changes with standard error compared to untreated controls after normalization with GAPDH mRNA expressions. Asterisks indicated statistically significant differences (*p*-value<0.05).

The mRNA level expressions of antiviral cytokines, such as IFN-α2, IFN-β1, IFN-γ, and pro-inflammatory cytokine IL-6 were observed to be not significantly different in fold change compared to mock-infected controls (Fig 5). However, the mRNA expression of TNF-α underwent significant up-regulation at 48 hpi compared to that of the mock-infected groups (Fig 5). It should be noted that at this time point, the virus titer in the supernatant was observed to reach the maximum titer during the viral growth kinetic study (Fig 4). These results suggested that H5N1 virus may have some mechanism to interfere with the acute IFN-mediated antiviral responses in infected cells, as suggested by a previous report [28].

### Phenotypic characterizations of hNPCs upon H5N1 infection

**Fig 6:**
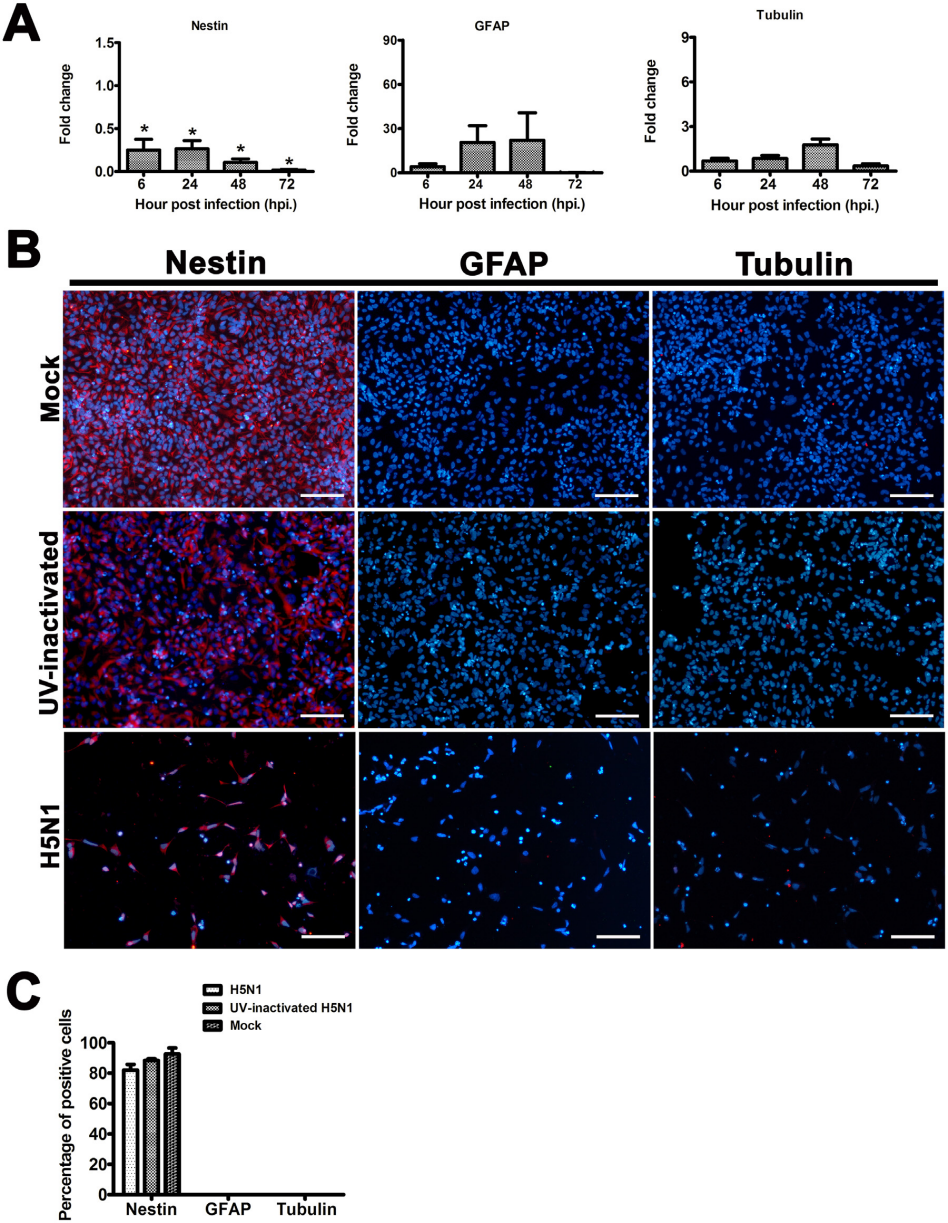
The differentiated phenotypic characterization of the hNPCs following infection with the H5N1, UV-inactivated H5N1, and mock-infected control. At 6–72 hpi, the expression of Nestin mRNA level was observed to be significantly down-regulated following virus infection, while the mRNA level expressions of β-III tubulin and GFAP were not found to show a significant difference compared to the mock infected control (**A**). Immunostaining of hNPCs at 72 hpi with antibodies against Nestin, GFAP and β-III tubulin in the H5N1-infected, UV-inactivated H5N1-infected, and mock-infected groups indicated that more than 80% of the cells were expressed Nestin, while stained negative for GFAP and β-III tubulin (**B, C**). Scale bar in B≈100 μm. Data are expressed as mean fold changes with standard error relative to mock-infected groups. Asterisks indicated statistically significant differences (*p*-value<0.05).

The mRNA level expressions determined by quantitative RT-PCR indicated that H5N1 virus infection significantly down-regulated the expression of Nestin, a specific marker for neural progenitors as early as 6 hpi (Fig 6A). However, the mRNA expressions of GFAP and β-III tubulin, cellular markers used to characterize astrocytic and neuronal development, respectively, were only slightly, but not statistically significantly increased (Fig 6A).

Immunophenotypic characterization of hNPCs at 72 hpi with H5N1 virus, UV-inactivated H5N1 virus and mock-infected control suggested that most of the cells were expressed Nestin, while they were negative for GFAP and β-III tubulin (Fig 6B). It should be noted that despite the marked decreased of cell numbers at 72 hpi in H5N1-infected groups, the percentages of Nestin-, GFAP-, and β-III tubulin-positive cells remained unchanged compared to that of UV-inactivated and mock-infected groups (Fig 6C), suggesting that either H5N1, or UV-inactivated H5N1 virus infection did not alter the phenotypes of the remaining living hNPCs.

## Discussion

Human neurodegenerative diseases, such as multiple sclerosis, encephalitis lethargica, Parkinson’s and Alzheimer’s diseases, have long been known for their significant pathological features of neuronal loss and damage in the CNS [29–31]. This is despite the fact that damaged neurons can be repaired by activation and differentiation of endogenous neural stem/progenitor cells residing in the SVZ of the lateral ventricles and the SGZ of the hippocampus [18,19]. These neural stem/progenitor cells can be classified *in vitro* or *in vivo* by stage-specific markers, such as NG2, Nestin, Pax6 and Sox1 [22,32]. The potential of these neural stem/progenitor cells to migrating to areas of damaged brain and differentiate into adult neurons has been hypothesized to be regulated by endogenous and exogenous factors within the environment of the lesions [32]. In patients with the above-mentioned neurodegenerative diseases, however, differentiation of these progenitor cells into mature neurons is usually limited, even though NG2-positive neural progenitor cells have been observed in or around the lesions [33–35]. The exact pathomechanisms underlying these neuronal losses and poor differentiation of neural progenitors remain unknown and subject to controversial discussion [31,36]. It has been postulated that inflammatory mediators secreted by other CNS tissues, such as microglia, astrocytes, and even the neural progenitor cells themselves may also play roles in this limited regeneration [37–39].

The present study demonstrated that hNPCs support H5N1 influenza virus replication *in vitro*. Although infections with the avian H5N1 virus of stem/progenitor cells from blood and bone marrow [40,41], or human neuronal and astrocytic cell lines [26], have been reported, the present study appears the first to describe the interaction of H5N1 virus with human NPCs. Infection of the H5N1 virus in human brain cells has been shown previously to bring about up-regulation of mRNA levels of pro- and anti-inflammatory cytokines [26]. In addition, infection of monocyte-derived human macrophages by influenza A viruses induced a significant up-regulation of pro- and anti-inflammatory cytokines, including TNF-α, IFN-α and IFN-β [42]. It is known that interferon response has some potent antiviral effects during H5N1 virus infection [43,44]. The impairment of interferon production caused by H5N1 infection of the hNPCs in the present study suggests an underlying role of H5N1 virus in acute neuroinflammation and neurodegeneration. Similarly to the previous findings, we observed that infection of hNPCs by H5N1 virus resulted in marked up-regulation of TNF-α mRNA expression [26]. Activation of TNF-α has been postulated to induce apoptosis and cell death of H5N1 infected cells [26]. Moreover, TNF-α seems to play a detrimental role in neural survival and differentiation [45]. Upregulation of TNF-α by microglial cells could contribute to necrosis of hippocampal progenitor cells [46]. Furthermore, up-regulation of TNF-α is important for disruption of the blood brain barrier (BBB) [47,48]. Such disruption results in leakage and accumulation of blood vessel fluid in the brain and spinal cord parenchyma, leading to CNS edema.

Infection of the neural progenitor cells by neurovirulent viruses such as Borna disease virus (BDV) has been shown *in vitro* to impair cellular development of progenitor cells [20]. A previous study also demonstrated that BDV infection decreases cell numbers during neural differentiation [20]. In the present study, cell death was observed predominantly in H5N1-infected groups, whereas the phenotype of the remaining hNPCs following H5N1 virus infection remained unaltered. These results could be explained by the facts that the differentiation protocol was not introduced in the present study. Moreover, the differentiation stage of these cells before cell death is unknown. Although we believe that the decrease in cell numbers were not due to experimental artifact, since the same number of cells were seeded into both infected- and mock-infected groups prior to virus infection. Though it is not known whether such an infection could induce differentiation of the hNPCs and, at the same time, triggers cell death of these differentiating cells. Still, it remains to be determined whether susceptible infection of H5N1 virus depends on the differentiation stages of hNPCs, or the differentiation capacity of hNPCs is triggered by H5N1 virus. Previous studies have shown that viruses can induce cellular dedifferentiation by either direct lytic effect or cellular dysregulation following exposure to pro- and inflammatory cytokines [20,21,39,49]. Taken together, the present study showed that hNPCs support H5N1 virus infection *in vitro*. Infection resulted in significant cell losses and up-regulation of TNF-α pro-inflammatory cytokine. Moreover, phenotypic characterization of hNPCs was unaltered following H5N1 virus infection. Although our data did not demonstrate the dysregulation of hNPC development during acute H5N1 virus infection, it remains to be determined whether H5N1 virus or other low pathogenic strains of influenza viruses can deregulate neurogenesis or astrogliogenesis during the acute and chronic phase of infection. The results of the present study further support the role of at least one type of influenza virus in inducing encephalopathy.

## References

[1] de Jong MD, Hien TT. Avian influenza A (H5N1). J Clin Virol. 2006; 35:2–13. 16213784 10.1016/j.jcv.2005.09.002 PMC7108344

[2] Peiris JS, Yu WC, Leung CW, Cheung CY, Ng WF, Nicholls JM, et al Re-emergence of fatal human influenza A subtype H5N1 disease. Lancet. 2004; 363:617–619. 14987888 10.1016/S0140-6736(04)15595-5 PMC7112424

[3] Yuen KY, Chan PK, Peiris M, Tsang DN, Que TL, Shortridge KF, et al Clinical features and rapid viral diagnosis of human disease associated with avian influenza A H5N1 virus. Lancet. 1998; 351:467–471. 9482437 10.1016/s0140-6736(98)01182-9

[4] WHO. Cumulative number of confirmed human cases for avian influenza A(H5N1) reported to WHO.2014.

[5] Horimoto T, Kawaoka Y. Pandemic threat posed by avian influenza A viruses. Clin Microbiol Rev. 2001; 14:129–149. 11148006 10.1128/CMR.14.1.129-149.2001 PMC88966

[6] Kitcharoen S, Pattapongsin M, Sawanyawisuth K, Angela V, Tiamkao S. Neurologic manifestations of pandemic (H1N1) 2009 virus infection. Emerg Infect Dis. 2010; 16:569–570. 10.3201/eid1603.091699 20202451 PMC3322043

[7] Chotpitayasunondh T, Ungchusak K, Hanshaoworakul W, Chunsuthiwat S, Sawanpanyalert P, Kijphati R, et al Human disease from influenza A (H5N1), Thailand, 2004. Emerg Infect Dis. 2005; 11:201–209. 15752436 10.3201/eid1102.041061 PMC3320461

[8] Ng WF, To KF. Pathology of human H5N1 infection: new findings. Lancet. 2007; 370:1106–1108. 17905148 10.1016/S0140-6736(07)61490-1

[9] Tran TH, Nguyen TL, Nguyen TD, Luong TS, Pham PM, Nguyen V, et al Avian influenza A (H5N1) in 10 patients in Vietnam. N Engl J Med. 2004; 350:1179–1188. 14985470 10.1056/NEJMoa040419

[10] Korteweg C, Gu J. Pathology, molecular biology, and pathogenesis of avian influenza A (H5N1) infection in humans. Am J Pathol. 2008; 172:1155–1170. 10.2353/ajpath.2008.070791 18403604 PMC2329826

[11] Ng WF, To KF, Lam WW, Ng TK, Lee KC. The comparative pathology of severe acute respiratory syndrome and avian influenza A subtype H5N1—a review. Hum Pathol. 2006; 37:381–390. 16564911 10.1016/j.humpath.2006.01.015 PMC7112039

[12] Claas EC, Osterhaus AD, van Beek R, De Jong JC, Rimmelzwaan GF, Senne DA, et al Human influenza A H5N1 virus related to a highly pathogenic avian influenza virus. Lancet. 1998; 351:472–477. 9482438 10.1016/S0140-6736(97)11212-0

[13] Kuiken T, Taubenberger JK. Pathology of human influenza revisited. Vaccine. 2008; 26:D59–66 19230162 10.1016/j.vaccine.2008.07.025 PMC2605683

[14] Klopfleisch R, Werner O, Mundt E, Harder T, Teifke JP. Neurotropism of highly pathogenic avian influenza virus A/chicken/Indonesia/2003 (H5N1) in experimentally infected pigeons (*Columbia livia f*. *domestica*). Vet Pathol. 2006; 43:463–470. 16846988 10.1354/vp.43-4-463

[15] Vascellari M, Granato A, Trevisan L, Basilicata L, Toffan A, Milani A, et al Pathologic findings of highly pathogenic avian influenza virus A/Duck/Vietnam/12/05 (H5N1) in experimentally infected pekin ducks, based on immunohistochemistry and in situ hybridization. Vet Pathol. 2007; 44:635–642. 17846235 10.1354/vp.44-5-635

[16] Zhang Z, Zhang J, Huang K, Li KS, Yuen KY, Guan Y, et al Systemic infection of avian influenza A virus H5N1 subtype in humans. Hum Pathol. 2009; 40:735–739. 10.1016/j.humpath.2008.08.015 19121843 PMC7112124

[17] Jang H, Boltz D, Sturm-Ramirez K, Shepherd KR, Jiao Y, Webster R, et al Highly pathogenic H5N1 influenza virus can enter the central nervous system and induce neuroinflammation and neurodegeneration. Proc Natl Acad Sci U S A. 2009; 106:14063–14068. 10.1073/pnas.0900096106 19667183 PMC2729020

[18] Curtis MA, Low VF, Faull RL. Neurogenesis and progenitor cells in the adult human brain: a comparison between hippocampal and subventricular progenitor proliferation. Dev Neurobiol. 2012; 72:990–1005. 10.1002/dneu.22028 22539366

[19] Gage FH, Temple S. Neural stem cells: generating and regenerating the brain. Neuron. 2013; 80:588–601. 10.1016/j.neuron.2013.10.037 24183012

[20] Brnic D, Stevanovic V, Cochet M, Agier C, Richardson J, Montero-Menei CN, et al Borna disease virus infects human neural progenitor cells and impairs neurogenesis. J Virol. 2012; 86:2512–2522. 10.1128/JVI.05663-11 22190725 PMC3302287

[21] Dukhovny A, Sloutskin A, Markus A, Yee MB, Kinchington PR, Goldstein RS. Varicella-zoster virus infects human embryonic stem cell-derived neurons and neurospheres but not pluripotent embryonic stem cells or early progenitors. J Virol. 2012; 86:3211–3218. 10.1128/JVI.06810-11 22238301 PMC3302301

[22] Rungsiwiwut R, Manolertthewan C, Numchaisrika P, Ahnonkitpanit V, Virutamasen P, Techakumphu M, et al The ROCK inhibitor Y-26732 enhances the survival and proliferation of human embryonic stem cell-derived neural progenitor cells upon dissociation. Cells Tissues Organs. 2013; 198:127–138. 10.1159/000354031 24158103

[23] Wang G, Zhang J, Li W, Xin G, Su Y, Gao Y, et al Apoptosis and proinflammatory cytokine responses of primary mouse microglia and astrocytes induced by human H1N1 and avian H5N1 influenza viruses. Cell Mol Immunol. 2008; 5:113–120. 10.1038/cmi.2008.14 18445341 PMC4651245

[24] Viseshakul N, Thanawongnuwech R, Amonsin A, Suradhat S, Payungporn S, Keawchareon J, et al The genome sequence analysis of H5N1 avian influenza A virus isolated from the outbreak among poultry populations in Thailand. Virology. 2004; 328:169–176. 15464837 10.1016/j.virol.2004.06.045

[25] Pringproa K, Rohn K, Kummerfeld M, Wewetzer K, Baumgartner W. Theiler's murine encephalomyelitis virus preferentially infects immature stages of the murine oligodendrocyte precursor cell line BO-1 and blocks oligodendrocytic differentiation *in vitro* . Brain Res. 2010; 1327:24–37. 10.1016/j.brainres.2010.02.068 20206147

[26] Ng YP, Lee SM, Cheung TK, Nicholls JM, Peiris JS, Ip NY. Avian influenza H5N1 virus induces cytopathy and proinflammatory cytokine responses in human astrocytic and neuronal cell lines. Neuroscience. 2010; 168:613–623. 10.1016/j.neuroscience.2010.04.013 20398740

[27] Lehmann U, Kreipe H. Real-time PCR analysis of DNA and RNA extracted from formalin-fixed and paraffin-embedded biopsies. Methods. 2001; 25:409–418. 11846610 10.1006/meth.2001.1263

[28] Matthaei M, Budt M, Wolff T. Highly pathogenic H5N1 influenza A virus strains provoke heterogeneous IFN-alpha/beta responses that distinctively affect viral propagation in human cells. PLoS One. 2013; 8:e56659 10.1371/journal.pone.0056659 23451066 PMC3581526

[29] Dale RC, Church AJ, Surtees RA, Lees AJ, Adcock JE, Harding B, et al Encephalitis lethargica syndrome: 20 new cases and evidence of basal ganglia autoimmunity. Brain. 2004; 127:21–33. 14570817 10.1093/brain/awh008

[30] Dickman MS. von Economo encephalitis. Arch Neurol. 2001; 58:1696–1698. 11594935 10.1001/archneur.58.10.1696

[31] Hardy J, Orr H. The genetics of neurodegenerative diseases. J Neurochem. 2006; 97:1690–1699. 16805777 10.1111/j.1471-4159.2006.03979.x

[32] Bellenchi GC, Volpicelli F, Piscopo V, Perrone-Capano C, di Porzio U. Adult neural stem cells: an endogenous tool to repair brain injury? J Neurochem. 2013; 124:159–167. 10.1111/jnc.12084 23134340

[33] Penderis J, Shields SA, Franklin RJ. Impaired remyelination and depletion of oligodendrocyte progenitors does not occur following repeated episodes of focal demyelination in the rat central nervous system. Brain. 2003; 126:1382–1391 12764059 10.1093/brain/awg126

[34] Kuhlmann T, Miron V, Cui Q, Wegner C, Antel J, Bruck W. Differentiation block of oligodendroglial progenitor cells as a cause for remyelination failure in chronic multiple sclerosis. Brain. 2008; 131:1749–1758. 10.1093/brain/awn096 18515322

[35] Aponso PM, Faull RL, Connor B. Increased progenitor cell proliferation and astrogenesis in the partial progressive 6-hydroxydopamine model of Parkinson's disease. Neuroscience. 2008; 151:1142–1153. 10.1016/j.neuroscience.2007.11.036 18201835

[36] Morris M, Koyama A, Masliah E, Mucke L. Tau reduction does not prevent motor deficits in two mouse models of Parkinson's disease. PLoS One. 2011; 6:e29257 10.1371/journal.pone.0029257 22206005 PMC3242771

[37] Lan X, Chen Q, Wang Y, Jia B, Sun L, Zheng J, et al TNF-alpha affects human cortical neural progenitor cell differentiation through the autocrine secretion of leukemia inhibitory factor. PLoS One. 2012; 7:e50783 10.1371/journal.pone.0050783 23236394 PMC3517586

[38] Wang FW, Hao HB, Zhao SD, Zhang YM, Liu Q, Liu HJ, et al Roles of activated astrocyte in neural stem cell proliferation and differentiation. Stem Cell Res. 2011; 7:41–53. 10.1016/j.scr.2011.03.004 21530437

[39] Cacci E, Ajmone-Cat MA, Anelli T, Biagioni S, Minghetti L. *In vitr*o neuronal and glial differentiation from embryonic or adult neural precursor cells are differently affected by chronic or acute activation of microglia. Glia. 2008; 56:412–425. 10.1002/glia.20616 18186084

[40] Khatri M, Saif YM. Epithelial cells derived from swine bone marrow express stem cell markers and support influenza virus replication *in vitro* . PLoS One. 2011; 6:e29567 10.1371/journal.pone.0029567 22216319 PMC3245290

[41] Thanunchai M, Kanrai P, Wiboon-Ut S, Puthavathana P, Hongeng S, Thitithanyanont A. Tropism of avian influenza A (H5N1) virus to mesenchymal stem cells and CD34+ hematopoietic stem cells. PLoS One. 2013; 8:e81805 10.1371/journal.pone.0081805 24339969 PMC3858287

[42] Lee DC, Law AH, Hui K, Tam AH, Peiris JS, Lau AS. Interferon dysregulation and virus-induced cell death in avian influenza H5N1 virus infections. Hong Kong Med J. 2012; 18:12–16 22311354

[43] Abdel-Ghafar AN, Chotpitayasunondh T, Gao Z, Hayden FG, Nguyen DH, de Jong MD, et al Update on avian influenza A (H5N1) virus infection in humans. N Engl J Med. 2008; 358:261–273. 10.1056/NEJMra0707279 18199865

[44] Guo Z, Chen LM, Zeng H, Gomez JA, Plowden J, Fujita T, et al NS1 protein of influenza A virus inhibits the function of intracytoplasmic pathogen sensor, RIG-I. Am J Respir Cell Mol Biol. 2007; 36:263–269. 17053203 10.1165/rcmb.2006-0283RC

[45] Liu YP, Lin HI, Tzeng SF. Tumor necrosis factor-alpha and interleukin-18 modulate neuronal cell fate in embryonic neural progenitor culture. Brain Res. 2005; 1054:152–158. 16054598 10.1016/j.brainres.2005.06.085

[46] Vezzani A, Moneta D, Richichi C, Aliprandi M, Burrows SJ, Ravizza T, et al Functional role of inflammatory cytokines and antiinflammatory molecules in seizures and epileptogenesis. Epilepsia. 2002; 43:30–35. 12121291 10.1046/j.1528-1157.43.s.5.14.x

[47] de Vries HE, Blom-Roosemalen MC, van Oosten M, de Boer AG, van Berkel TJ, Breimer DD, et al The influence of cytokines on the integrity of the blood-brain barrier in vitro. J Neuroimmunol. 1996; 64:37–43 8598388 10.1016/0165-5728(95)00148-4

[48] Kitic M, Hochmeister S, Wimmer I, Bauer J, Misu T, Mader S, et al Intrastriatal injection of interleukin-1 beta triggers the formation of neuromyelitis optica-like lesions in NMO-IgG seropositive rats. Acta Neuropathol Commun. 2013; 1:5 10.1186/2051-5960-1-5 24252536 PMC3776214

[49] Das S, Basu A. Viral infection and neural stem/progenitor cell's fate: implications in brain development and neurological disorders. Neurochem Int. 2011; 59:357–366. 10.1016/j.neuint.2011.02.020 21354238

